# Overexpression of Insig-1 protects β cell against glucolipotoxicity via SREBP-1c

**DOI:** 10.1186/1423-0127-18-57

**Published:** 2011-08-16

**Authors:** Ke Chen, ping jin, Hong-hui He, Yan-hong Xie, Xiao-yun Xie, Zhao-hui Mo

**Affiliations:** 1 Department of Endocrinology, Third Xiangya Hospital of Central South University, Changsha, China

## Abstract

**Background:**

High glucose induced lipid synthesis leads to β cell glucolipotoxicity. Sterol regulatory element binding protein-1c (SREBP-1c) is reported to be partially involved in this process. Insulin induced gene-1 (Insig-1) is an important upstream regulator of Insig-1-SREBPs cleavage activating protein (SCAP)-SREBP-1c pathway. Insig-1 effectively blocks the transcription of SREBP-1c, preventing the activation of the genes for lipid biosynthesis. In this study, we aimed to investigate whether Insig-1 protects β cells against glucolipotoxicity.

**Methods:**

An Insig-1 stable cell line was generated by overexpression of Insig-1 in INS-1 cells. The expression of Insig-1 was evaluated by RT-PCR and Western blotting, then, cells were then treated with standard (11.2 mM) or high (25.0 mM) glucose for 0 h, 24 h and 72 h. Cell viability, apoptosis, glucose stimulated insulin secretion (GSIS), lipid metabolism and mRNA expression of insulin secretion relevant genes such as IRS-2, PDX-1, GLUT-2, Insulin and UCP-2 were evaluated.

**Results:**

We found that Insig-1 suppressed the high glucose induced SREBP-1c mRNA and protein expression. Our results also showed that Insig-1 overexpression protected β cells from ER stress-induced apoptosis by regulating the proteins expressed in the IRE1α pathway, such as p-IRE1α, p-JNK, CHOP and BCL-2. In addition, Insig-1 up-regulated the expression of IRS-2, PDX-1, GLUT-2 and Insulin, down-regulated the expression of UCP-2 and improved glucose stimulated insulin secretion (GSIS). Finally, we found that Insig-1 inhibited the lipid accumulation and free fatty acid (FFA) synthesis in a time-dependent manner.

**Conclusions:**

There results suggest that Insig-1 may play a critical role in protecting β cells against glucolipotoxicity by regulating the expression of SREBP-1c.

## Background

Pancreatic β cell dysfunction is a crucial pathological contributor to the development of type 2 diabetes. The effects of glucose and free fatty acid (FFA) on β cell dysfunction have been extensively studied [1-4]. Chronic exposure to high glucose or high lipid leads to "glucotoxicity" or "lipotoxicity" [5]. The term "glucolipotoxicity" is now widely accepted to describe the combined effects of high glucose and high lipid on β cell dysfunction [6]. Apoptosis, impaired glucose stimulated insulin secretion (GSIS) [7] and lipid accumulation [8] are critical components involved in glucolipotoxicity.

Sterol regulatory element binding protein-1c (SREBP-1c), a lipogenic transcription factor, has been found to play a critical role in the development of β cell dysfunction caused by elevated glucose and FFA [9]. SREBP-1c is a membrane-bound transcription factor from the basic helix-loop-helix (bHLH) leucine zipper family and has been described as a regulator of lipogenic enzymes in liver, adipocytes, myocytes and β cells [10]. Overexpression of SREBP-1c induced β cell dysfunction, such as apoptosis, GSIS and lipid accumulation [9,11]. SREBP-1c preferentially activates genes involved in FFA and triglyceride synthesis, like fatty acid synthesis (FAS), elongation of very long-chain fatty acids (ELOVL), and Δ5-desaturase (DSR5) [12,13]. First, high glucose up-regulates the synthesis of FFA and leads to β cell apoptosis. Several mechanisms are implicated in this process, such as ER stress, oxidative stress, ceramide formation and modulation of microRNAs pathways [14-17]. ER stress mechanism is When FFA activates misfolded proteins in the ER lumen, Igheavy chain binding protein (BIP) dissociates from ER stress transducers. BIP then leads to an unfolded protein respond (UPR), including inositol requiring ER-to-nucleus signal kinase 1α (IRE1α), activating transcription factor (ATF6), and PKR-like ER kinase (PERK), the UPR is activated. The IRE1α then activates c-Jun N-terminal kinase (JNK), C/EBP homologous protein (CHOP), inhibits BCL-2 and results in apoptosis [18]. Second, increased FFA synthesis results in impaired GSIS. SREBP-1c is implicated to be involved through regulation of insulin receptor substrate 2 (IRS-2), pancreatic duodenal homeobox factor-1 (PDX-1) and uncoupling protein-2 (UCP-2) [19]. SREBP-1c directly regulates the transcription of the genes mentioned above and glucose transporter isoform-2 (GLUT-2) through sterol response element (SRE). GLUT-2 takes up glucose thereby increases ATP and ultimately up-regulates the expression of insulin [8]. Third, SREBP-1c regulates lipid synthesis in β cell. High glucose both acutely and chronically induces de novo lipogenesis, and activates SREBP-1c transcription of lipid synthesis [20].

Insulin induced gene-1 (Insig-1), an ER-resident protein that contains six transmembrane segments, negatively regulates SREBPs and HMG-CoA reductase, and plays a critical role in the feedback control of lipid synthesis. Sterol-stimulated binding of Insig-1 to SREBPs cleavage activating protein (SCAP) facilitates the retention of SCAP/SREBP-1c complex in the ER, prevents SREBP-1c from entering into the Golgi apparatus, decreases production of the nuclear forms of SREBPs (nSREBPs) and limits transcription of SREBP-1c target genes [21]. Therefore, Insig-1 is an important upstream factor, which regulates lipid synthesis via Insig-1-SCAP-SREBP pathway. Overexpression of Insig-1 has been found to inhibit lipid synthesis both in vitro and in vivo experiments [22], however, it is still unclear whether Insig-1 regulates β cell function at SREBP-1c level.

The present study was performed to comprehensively investigate whether overexpression of Insig-1 protects β cells against glucolipotoxicity via SREBP-1c. Insig-1 stable transfected INS-1 cells were generated and the cell viability, apoptosis, insulin secretion, lipid metabolism and mRNA expression of relevant genes such as IRS-2, PDX-1, GLUT-2, Insulin and UCP-2 were evaluated.

## Materials and methods

### Plasmid construction

The mouse Insig-1 cDNA fragment was generated by RT-PCR. Total RNA was extracted from mouse 3T3-L1 cells using TRIzol Reagent (Invitrogen, USA) following manufacture's instructions. Total RNA was reverse transcribed using moloney murine leukemia virus reverse transcriptase (Fermentas, USA). Fragments of mouse Insig-1 (NM-153526.5) cDNA were amplified by PCR using the following primers: forward5'-GAAGCTTATGCCCAGGCTGC-3' and reverse 5'-GAATTCTTCCACTCTGAACCATGT-3'. Hind III and EcoR I restriction sites were included (underlined) to facilitate the ligation of cDNA product into pcDNA3.1(+) vector (Invitrogen, USA). The mouse Insig-1 primers used in this study were tested using Blast software (http://blast.ncbi.nlm.nih.gov/Blast.cgi), and did not recognize the rat Insig-1 mRNA sequence. The sequence of pcDNA3.1(+)-Insig-1 was verified by DNA sequencing.

### Cell culture and generation of stable transfection cell line

Rat pancreatic INS-1 cells were maintained in RPMI 1640 medium (Gibco-BRL, U.K.) with 11.2 mM glucose supplemented with 10% heat-inactivated fetal calf serum, 10 mM HEPES, 2 mM L-glutamine, 1 mM sodium pyruvate, 50 μΜ β-mercaptoethanol, 100 U/ml penicillin and 100 mg/ml streptomycin at 37°C in a humidified atmosphere (5% CO2 and 95% air).

To generate an Insig-1 overexpression INS-1 cell line (INS-1-Insig-1), INS-1 cells were transfected with pcDNA3.1(+)-Insig-1. In brief, INS-1 cells were plated in a six-well plate and grown overnight to approximately 60% confluency. Cells were transfected using Lipofectamine 2000 (Invitrogen, USA) and 1.6 μg DNA per well in serum-free medium according to the manufacturer's instructions. After 48 h, cells were subjected to 100 μg/ml G418 selection for 30 days. Stable transfection cell line was derived from a single stable clone. Insig-1 gene and protein expression were confirmed by RT-PCR and Western blot analysis, respectively.

To study the effects of Insig-1 on glucose-induced glucolipotoxicity on β cell, INS-1-Insig-1 cells and control INS-1 cells were incubated in RPMI medium at 11.2 mM or 25.0 mM glucose for 0, 24 and 72 h.

### MTT assay

Cell viability was measured by adding 200 g/ml 3-(4,5-Dimethylthiazol-2-yl)-2,5-diphenyltetrazolium bromide(MTT) (Dingguo, China) to INS-1 cells and INS-1-Insig-1 cells, and incubated for 3 h at 37°C. The reaction was stopped and the purple formazan precipitate formed was dissolved using dimethyl sulfoxide (DMSO) and the color intensity was measured at 550 nm using a multiwell spectrophotometer (Thermo Labsystems, USA).

### Detection of apoptotic cells

For flow cytometric analysis, cells were collected by trypsinization and washed with cold phosphate-buffered saline and incubated for 10 min with Annexin V (Sigma, USA) for 15 min, then stained with propidium iodide (PI) (Sigma, USA). The analysis was performed with a FACScan flow cytometer (BD Biosciences, USA) using the CellQuest software (BD Biosciences). Cells that are in early apoptosis are Annexin V positive and PI negative.

### Western blot analysis

Proteins were extracted as previously described [23,24]. Briefly, cells were washed twice with PBS, and protein was extracted using lysis buffer (Sigma, USA). The supernatant was obtained by centrifugation at 4°C, 12,000 rmp for 10 minutes. The nuclear protein was extracted using a nuclear protein extraction kit (Generay, China) according to the manufacture's protocol. Proteins were resolved by SDS-PAGE and were transferred to nitrocellulose membranes. After incubation with primary antibody and secondary antibody conjugated to horseradish peroxidase, the bands were detected with the enhanced chemiluminescence system (Amersham Bioscience, USA). Immunoblots were scanned and quantified using Scion Image software (Scion Corporation, USA). The following primary antibodies (Santa Cruz Biotechnology or Novus Biologicals, USA) and dilution were used: Insig-1 (sc-51102, 1:500), nSREBP-1 (sc-8984, 1:300), p-JNK(sc-6254, 1:100), CHOP (sc-575, 1:500), BCL-2 (sc-7382, 1:200), GAPDH (sc-47724, 1:200) and p-IRE1α (NB100-2323, 1:500).

### Glucose stimulated insulin secretion (GSIS)

After exposure to the indicated glucose (11.2 mM or 25.0 mM) for 0, 24 and 72 h, two groups of cells were washed twice with PBS, followed by pre-incubation in Krebs-Ringer bicarbonate HEPES buffer (KRBH buffer: 115 mM NaCl, 24 mM NaHCO3, 5 mM KCl, 1 mM MgCl2, 25 mM HEPES, 0.5% BSA, pH 7.4) containing 3 mM glucose at 37°C for 30 min. The buffer was removed completely and collected for basal insulin secretion measurement. Fresh KRBH buffer containing 20 mM glucose was then added to the cells and cells were incubated for an additional 30 min as glucose stimulated insulin secretion. Insulin concentration was measured using an insulin radioimmunoassay (RIA) kit (Beijing Atom HighTech, China) according to the manufacture's protocol.

### Total RNA preparation and real-time PCR

Total RNA was extracted and reverse transcribed as described above. Real-time PCR was performed using QuantiTect SYBR green PCR master mix kit (ToYoBo, Japan) following the manufacturer's instructions on a mastercycler EP realplex RT-PCR instrument (Eppendorf, Germany). The reaction volume was 10 μL and contained 5 μL QuantiTect SYBR green PCR master mix, 0.5 μmol/L primers and 100 ng cDNA and RNase-free water. Primer sequences used in the PCR are provided in Table 1. The PCR conditions were: an initial denaturation at 95°C for 1 min, followed by 40 PCR cycles. Each cycle consisted of 15 s at 95°C, 30 s at 60°C and 30 s at 72°C. All quantifications were performed with rat GAPDH as an internal standard. The relative amount of all mRNA was calculated using the 2-ΔΔCT method.

**Table 1:** Sequence information on the primers used for real-time PCR

Gene name	Sequences for forward and reverse primers (5'-3')	Gene Bank accession number
SREBP-1c	GGAGCCATGGATTGCACATT	L16995
	AGGCCAGGGAAGTCACTGTCT	
IRS-2	CCACACGCCTTTCGCTAGA	XM-573948
	GTACCCCCTTCACCAAAGTCAA	
UCP-2	GCATTGGCCTCTACGACTCT	NM-019354.2
	CTGGAAGCGGACCTTTACC	
PDX-1	AAACGCCACACACAAGGAGAA	NM-022852
	AGACCTGGCGGTTCACATG	
GLUT-2	CAGCTGTCTCTGTGCTGCTTGT	NM-012879
	GCCGTCATGCTCACATAACTCA	
Insulin	TCTTCTACACACCCATGTCCC	NM-019130
	GGTGCAGCACTGATCCAC	
FAS	GCTGCTGCTGTGGACCTCAT	X62888
	TTTATACTGGTCGGCGGCTC	
GAPDH	TGGTGGACCTCATGGCCTAC	XM-344448
	CAGCAACTGAGGGCCTCTCT	

### Oil Red O staining and measurement of FFA content

Cells were fixed with 10% (v/v) formalin and were stained with Oil Red O as described by Kuri-Harcuch and Green [25]. Lipid droplets were observed and photographed under a microscope (TE2000-E; Nikon). At the end of 0, 24 and 72 h incubation, media were collected and FFA content of each sample was determined using an ELISA kit (Uscnlife, China) according to the manufacturer's protocol.

### Statistical analysis

Data shown are means ± SE. Statistical significance of differences between two groups was determined using the Student's t-test. Groups of three or more were analyzed by one-way ANOVA. P value of less than 0.05 was considered significant.

## Results

### mRNA and protein expression of Insig-1 in INS-1 cells overexpressed with Insig-1

**Figure 1:**
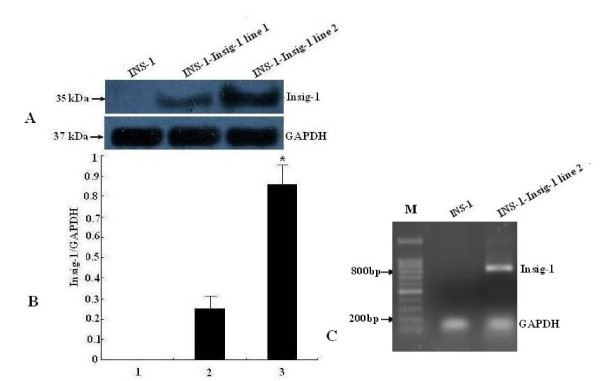
**Detection of Insig-1 mRNA and protein expression in INS-1-Insig-1 stable cells**. An Insig-1 stable cell line was generated by overexpression of Insig-1 in INS-1 cells, Insig-1 gene and protein expression were confirmed by RT-PCR and Western blot analysis, respectively. A) A mouse specific anti-Insig-1 primary antibody was used to detect mouse Insig-1 protein by Western blot analysis. GAPDH was used as control for protein loading. B) Relative expression of Insig-1 protein compared to GAPDH. 1, 2 and 3 represent control INS-1 cells, INS-1-Insig-1 cell line 1 and 2, respectively. *P < 0.05. C) Mouse Insig-1 mRNA expression was detected by RT-PCR, INS-1-Insig-1 cell line 2 is shown and used for further experiments. M: Marker.

To determine the transfection efficiency, we analyzed protein and mRNA expression of mouse Insig-1 in INS-1-Insig-1 cells. The Insig-1 primary antibody was mouse specific and did not cross react with rat Insig-1 protein. The Western blot results showed that Insig-1 protein was not expressed in control INS-1 cells, while mouse Insig-1 was highly expressed in two randomly selected stable cell lines with Insig-1 overexpression (sample 1 and 2), and the expression was especially high in sample 2 (Figure 1A, B). Similarly, mouse Insig-1 mRNA was not expressed in control INS-1 cells, but the 819 bp mouse Insig-1 fragment was detected in INS-1-Insig-1 stable cell lines (especially in sample 2) which was in accordance with our original design (Figure 1C). Sample 2 was thus selected as our INS-1-Insig-1 stable cell line for further experiment.

### Insig-1 increases cell viability and decreases apoptosis against glucolipotoxicity

**Figure 2:**
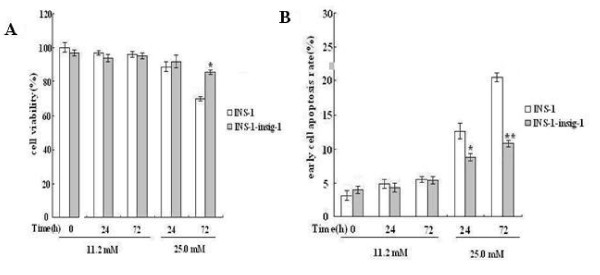
**Insig-1 increased cell viability and decreased cell apoptosis in the presence of high glucose induced glucolipotoxicity**. INS-1 and INS-1-Insig-1 cells were stimulated with 11.2 and 25.0 mM glucose for 0, 24 and 72 h. A) Cell viability was measured by MTT assay, B) Cell apoptosis rates were detected by flow cytometric analysis. *P < 0.05; **P < 0.01 VS INS-1 cell at the same time point.

To determine the effect of Insig-1 overexpression on cell viability and apoptosis, INS-1 cells and INS-1-Insig-1 cells were exposed to 11.2 and 25.0 mM glucose for 0, 24 and 72 h, respectively. The MTT assay results showed no difference in cell viability of those two groups at 11.2 mM glucose for all three time points. Interestingly, after 72 h incubation in 25.0 mM glucose, the cell viability of control INS-1 cells was significantly decreased by 30%, and decreased by only 14% in INS-1-Insig-1 cells (P < 0.05 vs INS-1 cells), (Figure 2A). Similar results were also detected by flow cytometric analysis, 24 h and 72 h treatment with 25.0 mM glucose caused a drastically increase of apoptosis in both control INS-1 cells, however, less apoptosis was found in INS-1-Insig-1 cells (13.1 ± 1.9% vs. 8.6 ± 2.1% for 24 h, 20.4 ± 2.5% vs. 10.9 ± 1.2% for 72 h, respectively, Figure 2B), suggesting that Insig-1 overexpression protected β cell viability and apoptosis against chronic high glucose induced glucolipotoxicity.

### Insig-1 suppresses nSREBP-1c expression and decreases ER stress through IRE1α pathway

**Figure 3:**
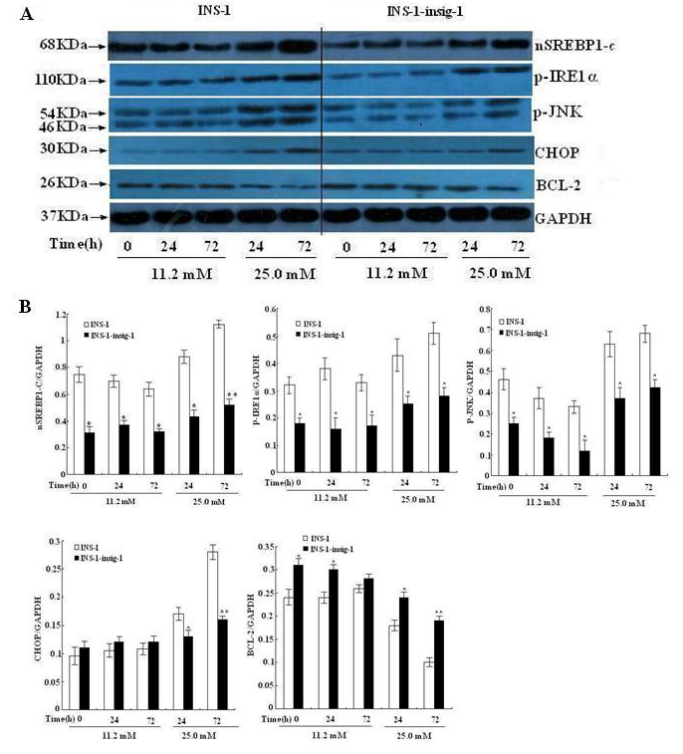
**Effect of Insig-1 overexpression on nSREBP-1c and proteins related to ER stress pathway in INS-1 cells**. INS-1 and INS-1-Insig-1 cells were stimulated with 11.2 mM and 25.0 mM glucose for 0, 24 and 72 h. A) nSREBP-1c and p-IRE1α pathway of ER stress related proteins were detected by Western blot. GAPDH was used as control for protein loading. Equal amount of proteins (50 μg) were loaded. B) Relative protein expression compared to GAPDH, data are the means ± SE of three independent experiments; *, P < 0.05, **, P < 0.01 VS INS-1 cells at the same time point.

To further investigate the underlying molecular mechanisms by which Insig-1 prevents β cell apoptosis, we examined the expression of nSREBP-1c, a nuclear active form of SREBP-1c. Cell total nuclear protein was extracted and SREBP-1c protein expression was evaluated by Western bolt analysis. The results showed that nSREBP-1c protein expression was significantly less up-regulated in control INS-1 cells exposed to 25.0 mM glucose for 24 h and 72 h, while in INS-1-Insig-1 cells, nSREBP-1c was significantly down-regulated when exposed to standard (11.2 mM) or high (25.0 mM) glucose compared to control INS-1 cells at all time points. We also examined ER stress related protein expression in IRE1α pathway. With 11.2 mM glucose stimulation, there was no change in CHOP expression between these two groups of cells at all three time points, while 25.0 mM glucose significantly decreased CHOP expression at 24 and 72 h in INS-1-Insig-1 cells compared to control INS-1 cells. The expression of p-IRE1α and P-JNK expression in INS-1-Insig-1 cells was significantly decreased compared to control INS-1 cells in every time points when exposed to standard (11.2 mM) or high (25.0 mM) glucose. However, BCL-2 expression in INS-1-Insig-1 cells was significantly increased compared to control INS-1 cells (Figure 3A, B). These results suggested that overexpression of Insig-1 down regulated the expression of nSREBP-1c, suppressed the IRE1α pathway of ER stress and prevented β cells from apoptosis.

### Insig-1 improved the GSIS in INS-1 cells

**Figure 4:**
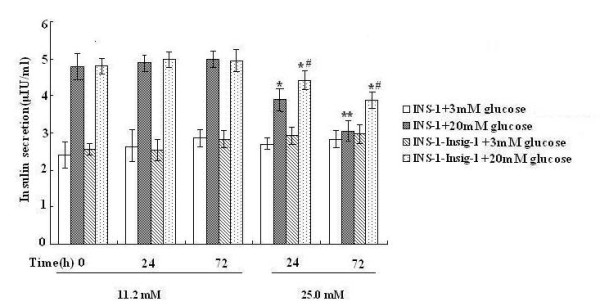
**Improvement of GSIS by Insig-1 overexpression**. INS-1 and INS-1-Insig-1 cells were cultured in RPMI 1640 with different glucose concentrations for 0, 24 and 72 h, medium were then changed to KRBH containing 3 mM and 20 mM glucose and then collected for basal and glucose stimulated insulin secretion. Insulin concentration was determined by rat insulin RIA. * P < 0.05; ** P < 0.01 VS exposed to 11.2 mM glucose at the same time point; # P < 0.05 VS INS-1 cells exposed to 25.0 mM glucose at the same time point.

To further investigate the effect of Insig-1 overexpression on insulin secretion, we measured the basal and GSIS in INS-1 and INS-1-Insig-1 cells. Cells were exposed to 11.2 or 25.0 mM glucose for 0, 24 and 72 h. There was no difference on basal insulin secretion between INS-1 and INS-1-Insig-1 cells after 11.2 mM or 25.0 mM glucose incubation for 0, 24 or 72 h. However, GSIS was markedly reduced (20.4% vs. 13.3% for 24 h, 39% vs. 21.7% for 72 h, respectively) after 24 and 72 h exposure to 25.0 mM glucose in both INS-1 and INS-1-Insig-1 cells. After 72 h stimulation with 25.0 mM glucose, the GSIS in control INS-1 cells decreased more dramatically compared with INS-1-Insig-1 cells (Figure 4). These results suggested that Insig-1 improved impaired GSIS induced by high glucose in INS-1 cells.

### Insig-1 regulates the expression of insulin secretion genes and FAS via SREBP-1c

**Figure 5:**
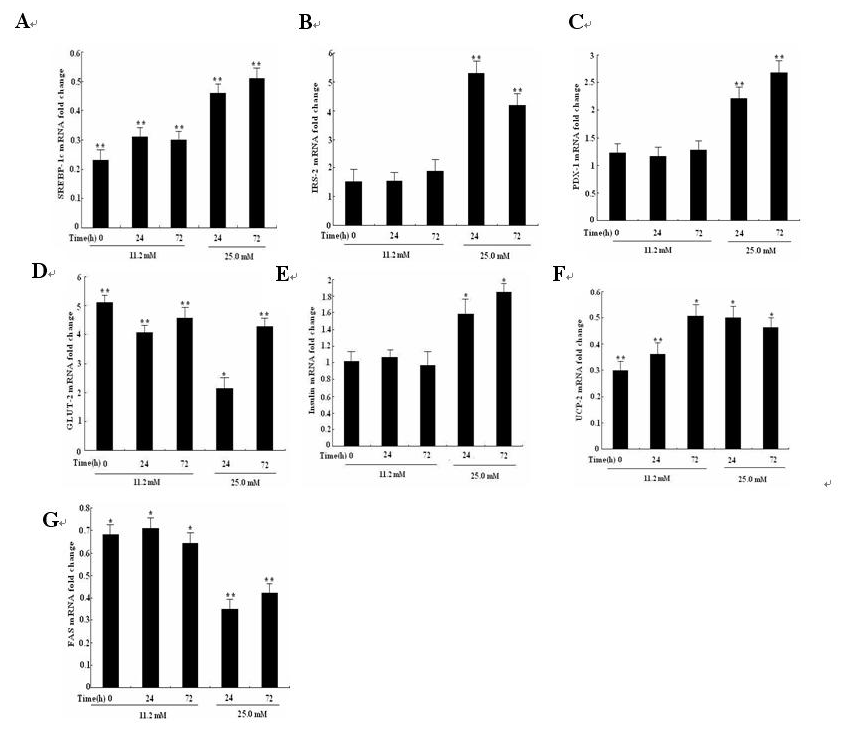
**Overexpression of Insig-1 suppressed SREBP-1c transcription and affected mRNA expression of insulin secretion genes**. Two groups of cells were exposed to 11.2 mM or 25.0 mM glucose for 0, 24 and 72 h, mRNA level of insulin secretion genes and FAS was detected by real-time PCR. A-G represented SREBP-1c, IRS-2, PDX-1, GLUT-2, insulin, UCP-2 and FAS mRNA fold change in INS-1-Insig-1 cells compared to INS-1 cells. * P < 0.05, ** P < 0.01 VS control INS-1 cells at the same time point.

To determine the molecular mechanisms by which Insig-1 improved insulin secretion and prevented the lipid synthesis, mRNA expression of insulin secretion related genes, such as IRS-2, PDX-1, GLUT-2, Insulin and UCP-2 were evaluated by real-time PCR. The results showed that SREBP-1c mRNA expression in both INS-1 and INS-1-Insig-1 cells was significantly up-regulated after 25.0 mM glucose stimulation, while in INS-1-Insig-1 cells, SREBP1-c mRNA was significantly down-regulated compare to that of control INS-1 cells upon treatment with 11.2 mM or 25.0 mM glucose and these effects were much prominent in 25.0 mM glucose at 72 h (Figure 5A). The insulin secretion related genes IRS-2, PDX-1 and GLUT-2 were also significantly up-regulated. When exposed to 11.2 mM or 25.0 mM glucose, IRS-2 mRNA level increased 1.52 - 5.31 fold (Figure 5B), PDX-1 mRNA level increased 1.23 - 2.86 fold (Figure 5C) and GLUT-2 mRNA level increased 2.18 - 5.10 fold at 0 h, 24 h and 72 h (Figure 5D), respectively. Although insulin mRNA expression was not changed by incubation with 11.2 mM glucose, it revealed a 1.59 and 1.82 fold increase with 25.0 mM glucose for 24 h and 72 h, respectively (Figure 5E). The mRNA level of UCP-2, a negative modulator of insulin secretion, decreased 49% -70% (Figure 5F). FAS is one of the most important lipid synthesis genes, the mRNA level of FAS decreased by 21% - 65% in INS-1- Insig-1 cells (Figure 5G). These results implicated that Insig-1 regulated insulin secretion and lipid genes expression through SREBP-1c.

### Insig-1 inhibits intracellular accumulation of lipid droplets and reduces FFA synthesis

**Figure 6:**
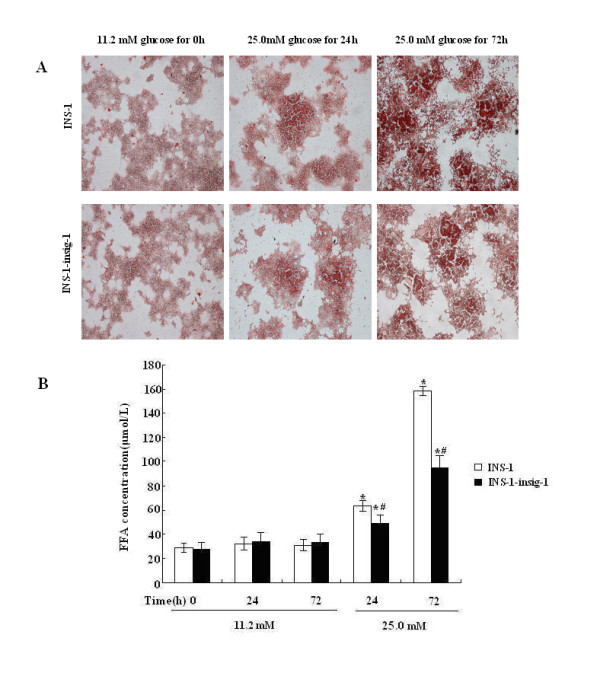
**Detection of intracellular lipid droplets in INS-1 cells by light microscopy and FFA measurement**. The INS-1 and INS-1-Insig-1 cells were exposed to 11.2 mM or 25.0 mM glucose for 0, 24 and 72 h. A) Oil Red O staining. The lipid droplets are stained dark red. (Magnification × 100) B) FFA concentration in the medium was measured by ELISA. * P < 0.05; ** P < 0.01 VS exposed to 11.2 mM glucose at the same time point; # P < 0.05 VS INS-1 cells at the same time point.

We further assessed whether Insig-1 prevented lipid accumulation and FFA synthesis. Intracellular lipid accumulation was evaluated by Oil Red O staining and light microscopy. FFA concentration was measured using ELISA. No lipid droplets were observed at 11.2 mM glucose in those two groups of cells (data of control INS-1 cells at 24 and 72 h were not shown), and few lipid droplets were detected following 25.0 mM glucose exposure for 24 h. Interestingly, a large amount of lipid droplets were accumulated in the cytoplasm by the end of 72 h following 25.0 mM glucose exposure. It should be noted that, INS-1-Insig-1 cells demonstrated less lipid droplets compared with control INS-1 cells (Figure 6A). No difference in FFA concentration was detected in the media of the cells exposed to 11.2 mM glucose, while FFA concentration was significantly increased by 25.0 mM glucose stimulation for 24 h and 72 h, and INS-1-Insig-1 cells showed less FFA concentration compared to control INS-1 cells (48.76 ± 5.64 vs. 63.50 ± 7.23 μmol/L in 24 h, 94.81 ± 4.12 vs. 158.43 ± 10.77 μmol/L in 72 h) (Figure 6B). These results indicated that high glucose induced FFA production, which leads to glucolipotoxicity in INS-1 cells, was attenuated by Insig-1 overexpression.

## Discussion

In the present study, we overexpressed Insig-1 in INS-1 cells and investigated the role of Insig-1 in glucolipotoxicity and demonstrated that Insig-1 partially improved β cell dysfunction induced by high glucose.

Glucolipotoxicity is an important factor in β cell dysfunction [26]. SREBP-1c is a critical component involved in this process and has been examined extensively. We hypothesized that the potential mechanism is that high glucose induces lipid accumulation through SREBP-1c and leads to β cell dysfunction, which is accompanied by apoptosis, impaired GSIS and lipid accumulation [9]. Overexpression of SREBP-1c has been found to induce glucolipotoxicity in insulinoma cells (INS-1 and MIN6) and isolated rat islets [27]. Insig-1 acts as an upstream regulator of SREBP-1c and regulates lipid metabolism through the Insig-1-SCAP-SREBP1-c pathway. In vitro experiments showed that overexpression of Insig-1 prevented lipogenesis and inhibited differentiation of preadipocyte 3T3-L1 cells [28]. Furthermore, an in vivo study using recombinant adenovirus containing mouse Insig-1 cDNA transfected Zucker diabetic fatty (ZDF) (fa/fa) rats resulted in a striking reduction of lipid synthesis in liver [29].

To investigate the underlying molecular mechanism of Insig-1 in preventing β cell apoptosis, we examined the expression of SREBP-1c mRNA and protein in control INS-1 cells and INS-1-Insig-1 cells under different incubation conditions. Both mRNA and protein expression of SREBP-1c were increased after 25.0 mM glucose stimulation in INS-1 and INS-1-Insig-1 cells, while INS-1-Insig-1 cells showed less SREBP-1c expression at all time points compared to control INS-1 cells, which is consistent with previous findings by others [27,28]. This suggests that overexpression of Insig-1 strongly suppresses SREBP-1c expression. We also assessed ER stress related protein expression in p-IRE1α pathway. ER stress plays an important role in β cell apoptosis. Wang et al. observed that the treatment of isolated rat islets with high glucose or ER stress inducers, drastically increased SREBP-1c activity, and the induction of a dominant negative mutant of SREBP-1c prevented high glucose induced ER stress [23]. Several studies have shown the p-IRE1α pathway which involves p-JNK activation, CHOP up-regulation and BCL-2 down-regulation in the process of apoptosis [29,30]. We observed that after exposing to high glucose, p-IRE1α, p-JNK and CHOP were markedly up-regulated, while BCL-2 were down-regulated in both INS-1 and INS-1-Insig-1 cells and Insig-1 overexpression cells showed less change compared with INS-1 cells. Thus, our study showed that overexpression of Insig-1 could strongly suppress p-IRE1α, p-JNK and CHOP protein expression after exposure to high glucose. At standard glucose level(11.2 mM), p-IRE1α and p-JNK protein expression were both down-regulated in INS-1-Insig-1 cells compared with INS-1 cells at all time points, while the protein expression of BCL-2 in INS-1-Insig-1 cells showed up-regulation compared to control INS-1 cells, though there was no difference in CHOP expression. We therefore postulated that even normal glucose concentrations could produce FFA to switch on the IRE1α pathway at this time.

SREBP-1c participates in multiple regulatory mechanisms of GSIS. In vitro, overexpression of SREBP-1c results in impaired insulin secretion in isolated rat islets [31]. In vivo, similar results were also observed by Takahashi et al. by using transgenic mice overexpressing the active form of SREBP-1c [32]. In addition, SREBP-1c knockout mice had increased basal and high glucose stimulated insulin secretion [33]. SREBP-1c also regulates the expression of insulin genes [34]. Recently, it was demonstrated that IRS-2, a gene plays an important role in β cell growth and survival, participated in GSIS through direct binding of SREBP-1c to its promoter [35]. Furthermore, both in vitro and in vivo experiments showed that overexpression of SREBP-1c could suppress the expression of PDX-1, a crucial transcription factor of insulin secretion [36]. It was also observed that high nutrition could accumulate the expression of UCP-2, a regulator of cytoplasmic ATP/ADP ratio in the process of GSIS, through SREBP-1c [34]. GLUT-2 is known as a transporter of glucose, and is activated upon binding with SREBP-1c [37]. Our results showed that in INS-1-Insig-1 cells, IRS-2, PDX-1 and GLUT-2 mRNA expressions were increased to various degrees compared with control INS-1 cells. The change in UCP-2 mRNA expression was similar to and in accordance with that of SREBP-1c, and although drastically increased at 25.0 mM glucose. Insulin mRNA expression did not increase when exposed to standard 11.2 mM glucose stimulation. These results are consistent with our mRNA study indicating that overexpression of Insig-1 decreased insulin secretion only after exposure to high glucose and we therefore postulated that other mechanisms might also be involved.

We measured the FFA concentration to further elucidate whether Insig-1 could suppress the lipid accumulation and FFA synthesis. Wang et al. [26] confirmed that chronic exposure to high glucose for 72 h induced lipid accumulation in INS-1 cells by increasing lipogenic gene expression, while chronic incubation with 1.5 mM FFA (2:1 oleate/palmitate) did not obtain the same results [38]. Our results showed a significantly increase of lipid droplets accumulation and FFA concentration after exposing to 25.0 mM glucose for 24 h and 72 h. We also verified that chronic high glucose induced FFA synthesis participated in β cell glucolipotoxicity, and this process was partially prevented by overexpression of Insig-1. FFA concentration and lipid accumulation in INS-1-Insig-1 cell were significantly decreased compare to that of control INS-1 cells.

We observed more drastically effect when two groups cultured for 72 h than 24 h, especially when INS-1 cells exposed to 25.0 mM glucose, for example, enhanced early cell apoptosis rate, an ER stress response, an inhibition of GSIS, an accumulation of intracellular lipid droplets and so on. We therefore postulate that high glucose stimulates more FFA, leads to lipotoxicity, and subsequently induces the impairment of beta cells.

## Conclusion

In summary, our results demonstrated that β cell dysfunction through chronic exposure of high glucose could be inhibited by Insig-1. The process may involve reducing cell apoptosis and increasing cell viability, preventing lipid accumulation and improving impaired GSIS. One possible molecular mechanism is that Insig-1 suppresses high glucose induced SREBP-1c transcription, which leads to reduced FFA and other lipid production, decreases expression of ER stress pathway protein and up-regulates the expression of insulin secretion genes. In conclusion, overexpression of Insig-1 protects β cells against glucolipotoxicity via SREBP-1c, thus improvement of Insig-1 activity should be considered as a therapy aimed at β cell protection.

## Competing interests

The authors declare that they have no competing interests.

## Authors' contributions

ZHM conceived the study, designed experiments. Experiments were performed by KC, PJ and HHH. Analysis of the data was performed by YHX, XYX. KC drafted the manuscript and all authors read and approved the final version.
